# Feasibility of using Arabic Hospital Anxiety and Depression Scale (HADS) to assess anxiety and depression among patients attending Accident and Emergency at a University Hospital setting in Riyadh, Saudi Arabia

**DOI:** 10.12669/pjms.316.6849

**Published:** 2015

**Authors:** Hosam A. Hassan, M. Owais Suriya, Zohair Ahmed Al-Aseri, Mujtaba Hasan, Najeeb Khalid, Shaffi Ahmed Sheikh

**Affiliations:** 1 Hosam A. Hassan, MBBCh, MRCPI. Assistant Professor and Consultant, Department of Emergency Medicine (65), College of Medicine, King Khalid University Hospital, Riyadh, Saudi Arabia; 2 M. Owais Suriya, MBBS, DTCD, MRCP (UK), MRCGP (UK) FCEM (UK). Research Fellow College of Medicine, University of Saskatchewan. 107, Wiggins Road S7N 5E5, Saskatoon, Canada; 3 Zohair Ahmed Al-Aseri, FRCPc, EM & CC. Associate Professor, Department of Emergency Medicine (65), College of Medicine, King Khalid University Hospital, Riyadh, Saudi Arabia; 4 Mujtaba Hasan, MBBS, BSc, FCPS, FCCP, DTP Assistant Professor of Medicine, Consultant Physician, University College of Medicine, University of Lahore, Pakistan; 5 Najeeb Khalid, MBBS (PK), MD (UK). Consultant Psychiatrist & HonSr Lecturer, Cardiff & Vale University Health Board, Cardiff, United Kingdom; 6 Shaffi Ahmed Sheikh, Ph.D. Associate Professor, Dept. Of Family & Community Medicine, College of Medicine, King Khalid University Hospital, Riyadh, Saudi Arabia

**Keywords:** Anxiety, depression, HADS, Accident & Emergency (A &E)

## Abstract

**Objective:**

To evaluate the feasibility of using Arabic Hospital Anxiety and Depression Scale (HADS) to assess depression and anxiety among patients attending accident and emergency (A & E) at a University Hospital setting in Riyadh, Saudi Arabia.

**Methods:**

In this prospective observational study translated questionnaire of HADS was used for patients aged 18 years or above who presented to A & E at King Khalid University Hospital in Riyadh, Saudi Arabia. The study included 257 patients as per an agreed inclusion criteria. The study quantified depression and anxiety and its association with demographic and or illness related variables using SPSS.

**Results:**

Out of 257 participants, the dominant age group, ranged between 18-30 years (40.9%) with female participants (55.3%) outweigh the male among all. The overall occurrence of depression was 27.2% (95% Confidence Interval (CI): 21.8 % to 32.6%) and anxiety was 23% (17.8% to 28.2%CI). Marital, educational and economic status of participants, were statistically significantly associated (p<0.05) with the levels of anxiety whereas age, marital, education, economic and employment status were associated (p<0.05) with the levels of depression.

**Conclusion:**

In the A & E setting at University Hospital in Saudi Arabia, comorbid depression and anxiety is not uncommon as enumerated by using HADS. The identified cases could then be sent for appropriate psychiatric treatment promptly not only to improve quality of individual care but also to reduce the overall health care costs in local context.

## INTRODUCTION

About 450 million people suffer from mental disorders according to estimates given decade ago in WHO World Health Report 2001^1^ and account for 11% of the total Disability Adjusted Life Years (DALYs) lost due to all diseases and injuries in the world.^2^ The prevalence of many mental disorders including depression and anxiety has generally been found further higher among patients with physical health problems. These psychiatric comorbidity results in lower adherence to medical treatment, an increase in disability and mortality, and higher health care costs.^2^,^3^ However, these comorbidities are often under recognized and, thus, not always effectively treated. Increased awareness and comprehensive integrated management to depression and anxiety may alleviate the burden caused by these common psychiatric conditions on the individual, society and the health care services. Appropriate action is, therefore, needed to overcome barriers which prevent people from receiving appropriate integrated management. Lack of skills at non psychiatric level is paramount feature, too few doctors and nurses know how to recognize and properly treat mental disorders. Despite the high global life time prevalence rate of depression (12%) and anxiety (15%),^4^ there are no mental health training programs for health care professionals in 41% of countries in the world.^3^,^4^

In the Arab world the prevalence of psychiatric comorbidities is reportedly high^5^-^7^ with consistently low rate of detection by the attending physicians. Possible reasons could be lack of confidence in procedure for detection of psychiatric comorbidity, lack in their training in this context, and more professional focus on possible somatic disorder.^3^,^4^

The HADS is most frequently used assessment tool that was developed by Zigmond^8^ to identify depression and anxiety among patients in non-psychiatric care settings. It has been in use extensively worldwide in the form of translated versions in various languages^9^ Arabic.^5^,^7^,^10^

The Arabic version of HADS has been used in different groups of patients both in primary care and hospital settings in Arab countries.^5^-^7^,^10^ However, it has not yet been tested in more acute clinical settings for instance in A & E in Arabian countries.

The present study, therefore, aimed to screen patients attending the A & E in a tertiary care hospital depression and anxiety as psychiatric comorbidities besides the correlation of it with relevant demographic variables.

## METHODS

The present study, a prospective observational survey, was carried out between February to December 2012 at the A & E in King Khalid University Hospital, King Saud University, Riyadh, Saudi Arabia. Permitted Arabic version of HADS by the sole proprietor, i.e., GL assessment UK^11^ was used. This questionnaire consists of an anxiety subscale (HADS-A) and a depression subscale (HADS-D) with 14 intermingled items. Each item is rated on a four-point scale of 0-3, giving maximum scores of 21 for anxiety and depression respectively. Scores of 11 or more on either subscale are considered as significant ’case’ of psychological comorbidity, a score of 8-10 as borderline while a score of 7 or below is considered as normal. The same cut-offs have been used by the comparable studies eleswhere.^12^,^13^

### Data collection and statistical analysis

The printed Arabic translated questionnaire of HADS was provided to each patient who was 18 years of age or more, who could read and understand the questionnaire, whereas significantly medically unstable and known psychiatric patients were excluded in order to avoid discrepancies in interpretation of results. The purpose of the questionnaire was fully described to the patients; consent was duly signed by each participant and they were provided 30 minutes time to fill it. Total number of patients who received the questionnaire were 298 among which 257 responses were included and 41 responses excluded from the study either due to refusal (29 patients) or incomplete filling of the questionnaire (12 patients). The demographic data of the patients were recorded by the A & E physicians on duty including age, gender, marital status, educational status, economical status, presenting complaint and existing medical problems and previous admissions in the hospital or A & E visits. The data was analyzed using SPSS version 16.0 (Chicago, IIL, USA).^14^ Descriptive statistics (proportions) were used to quantify the categorical variables. Pearson Chi-square test for trend to observe the distribution of dichotomous study variables across the ordinal outcome variables (depression and anxiety levels). Spearman rank correlation was used to observe the correlation between ordinal study and outcome variables. A p-value <0.05 was used to assess the statistical significance and precision of estimates.

## RESULTS

Out of 257 patients, there were 105 (40.9%) in the age range of 18-30 years and 115 (44.7%) were males. Illiteracy was observed in 84 ((32.7%) patients, 25 (9.7%) had higher education whereas remaining 148 (57.6%) were with primary and secondary literacy. Married patients were 139 (54.1%) and unmarried were 84 (32.7%) whereas 34 (13.2%) were divorced and widowed. Depression was noticed in 27.2% (95% CI: 21.8 % to 32.6%) and anxiety in 23% (17.8% to 28.2%).

### Trend of Anxiety levels across study variables

Of all the study variables marital and economic status had significant statistical association with the level of anxiety (p<0.05). Among them married and divorced/widowed had high association as compared to unmarried subjects, where 28.1% and 26.5% were ‘cases’ among married and divorced/widowed subjects compared to 13.1% in unmarried subjects. The lower socio economic (40.6%) and middle socio economic status subjects (22.8%) had significantly higher anxiety compared to upper socioeconomic class subjects (13%).

Other variables, although, were not statistically significant but anxiety was more common (28.3%) in the age group of 31-60 among those who were never admitted (25.5%), medical related patients (18.8%), and those who had poor prognosis (36.4%). (Table-I)

**Table-I:** Association of Anxiety levels with study and illness variables.

Study Variables	Anxiety Levels			X^2^ -value/	p-value
	Normal	Border line	Case	p- value**	
*Age groups*					
14-30	58(55.2)	29(27.6)	18(17.1)	0.049	0.43
31-60	49(49.5)	22(22.2)	28(28.3)		
>60	29(54.7)	11(20.8)	12(24.5)		
*Gender*					
Male	69(60)	22(19.1)	24(20.9)	2.55	0.11
Female	67(47.5)	39(27.7)	35(24.8)		
*Marital Status*					
Married	77(55.4)	23(16.5)	39(28.1)	16.99	0.002*
Unmarried	48(57.1)	25(29.8)	11(13.1)		
Divorced/widow	11(32.3)	14(41.2)	9(26.5)		
*Educational Status*					
Illiterate	40(47.6)	18(21.4)	26(31)	-0.107	0.087
Primary	36(52.9)	13(19.1)	19(27.9)		
Secondary	48(60)	25(31.2)	7(8.8)		
Higher	12(48)	6(24)	7(28)		
*Employment*					
Housewife	38(45.8)	20(24.1)	25(30.1)	9.15	0.33
Student	32(52.5)	20(32.8)	9(14.8)		
Employed	41(61.2)	12(17.9)	14(20.9)		
Unemployed	15(51.7)	6(20.7)	8(27.6)		
Retired	10(58.8)	4(23.5)	3(17.6)		
*Economical Status*					
Upper	33(61.1)	14(25.9)	7(13)	0.178	0.004*
Middle	93(54.4)	39(22.8)	39(22.8)		
Lower	10(31.2)	9(28.1)	13(40.6)		
*Previous Admission*					
No	84(52.2)	36(22.4)	41(25.5)	0.68	0.41
Yes	52(54.2)	26(27.1)	18(18.8)		
*Specialty*					
Medicine & Allied	46(54.1)	23(27.1)	16(18.8)	0.61	0.43
Surgical & allied	7(63.6)	3(27.3)	1(9.1)		
*Current admission*					
No	119(52.9)	55(24.3)	52(23)	0.02	0.88
Yes	15(53.6)	7(25)	6(21.4)		
*Progress*					
Improving	67(59.3)	24(21.2)	22(19.5)	4.25	0.37
Worsening	5(45.5)	2(18.2)	4(36.4)		
Variable	64(48.1)	36(27.1)	33(24.4)		
*Prognosis*					
Good	105(54)	44(21.8)	49(24.3)	0.044	0.83
Bad	27(49.1)	18(32.7)	10(18.2)		

* Statistically significant;

** Spearman correlation coefficient

### Trend of Depression levels across study variables

Of all the variables age, marital, educational, employment and economic status were statistically significantly associated with the level of depression (p<0.05). That is high proportion among patients with higher age group(47.20%), married (32.8%), divorced/widowed (41.2%), illiterate (39.2%), housewife (36.6%), unemployed (28.6%), retired(58.8%) and lower socio economic status(53.1%) compared to patients of lower age (17.1%), unmarried (13.1%), secondary (19%) and higher education (16%), employed (17.9%) and upper economic status(16.7%).

Other variables, although, were not statistically significant but it is worth mentioning here that depression was found more common among females(27.9%), those who never admitted (30.2%), among medical patients(22.4%) and among those who have poor prognosis (34.5%). (Table-II)

**Table-II:** Association of Depression levels with study and illness variables.

Study Variables	Depression levels			X^2^ -value/	p-value
	Normal	Mood disorder	Case	p- value**	
*Age groups*					
14-30	58(55.2)	29(27.6)	18(17.10)	0.24	<0.0001*
31-60	41(42.3)	29(29.9)	27(27.8)		
>60	16(30.2)	12(22.9)	25(47.20)		
*Gender*					
Male	55(48.2)	28(24.7)	31(27.2)	0.33	0.56
Female	60(42.9)	41(29.3)	39(27.9)	0.33	0.56
*Marital Status*					
Married	59(43.1)	33(24.1)	45(32.8)	17.92	0.0001*
Unmarried	48(57.1)	25(29.8)	11(13.1)		
Divorced/widow	8(23.5)	12(35.3)	14(41.2)		
*Educational Status*					
Illiterate	26(31.0)	25(29.8)	33(39.2)	-0.208	0.001*
Primary	35(52.2)	14(20.9)	18(26.9)		
Secondary	40(50.6)	24(10.4)	15(19)		
Higher	14(56)	7(28)	4(16)		
*Employment*					
Housewife	31(37.8)	21(25.6)	30(36.6)	21.7	0.006*
Student	35(57.4)	16(26.2)	10(16.4)		
Employed	31(46.3)	24(35.8)	12(17.9)		
Unemployed	12(42.9)	8(28.6)	8(28.6)		
Retired	6(30.3)	1(5.9)	10(58.8)		
*Economical Status*					
Upper	30(55.6)	10(27.8)	9(16.7)	0.215	0.001*
Middle	78(46.2)	47(27.8)	44(26)		
Lower	7(21.9)	8(25)	17(53.1)		
*Previous Admission*					
No	71(44.7)	40(25.2)	48(30.2)	0.62	0.43
Yes	44(45.8)	30(31.2)	22(22.9)		
*Specialty*					
Medicine & Allied	40(47.1)	26(30.6)	19(22.4)	0.37	0.54
Surgical & allied	4(36.4)	4(36.4)	3(27.3)		
*Current admission*					
No	101(45.1)	62(27.7)	61(27.2)	0.046	0.83
Yes	13(46.4)	4(36.4)	3(27.3)		
*Progress*					
Improving	58(52.3)	31(27.9)	22(19.8)	7.45	0.28
Worsening	3(27.3)	3(27.3)	5(45.5)		
Variable	54(40.6)	36(27.1)	43(31.3)		
*Prognosis*					
Good	95(47.5)	54(27)	51(25.5)	2.53	0.11
Bad	20(36.4)	16(29.1)	19(34.5)	2.53	0.11

* Statistically significant;

** Spearman correlation coefficient.

## DISCUSSION

Mental disorders will be the second leading cause of global disease burden by 2020^1^ with psychiatric co-morbidities still a leading cause of disability worldwide, both in community and in clinical population.^2^,^4^ Despite this enormous burden, physicians in non-psychiatric clinical settings merely predict comorbid psychiatric problems and, thus, patients often remain untreated. Prompt appropriate intervention for common psychiatric conditions, arguably, can improve treatment adherence and reduce health care costs by 25%, besides improving health care experience of patients.^3^ In clinical settings, A & E is the most hectic and often psychologically stressful setting. Despite the availability of this cost effective, reliable screening scale diagnosis of depression and anxiety is significantly low in acute settings with high reported failure in screening cases.^15^ This is probably due to very hectic working environment in hospital A & E and limited time for staff to consider psychiatric ailments among patients present with acute medical conditions arguably warrants priority to rescue serious acute presenting complaints. It has been suggested that unmet psychological needs in patients can complicate physical recovery in the short-term and negatively impact on patient compliance to follow-up care plan in the long-term.^16^,^17^ Therefore, screening of common psychiatric co-morbidities to be prioritized in clinical practice in health care disciplines^18^ thus, logically more vital in acute medical settings as A & E.^17^

The utility of a screening instrument in practice is determined by its case-finding ability. HADS evaluates reliably both depression and anxiety yet remains quick in time and straightforward to complete.^18^,^19^ It has been widely used by non-psychiatric physicians in various health care settings and in general population.^20^ It predicts the presence of depression and anxiety with a high degree of certainty.^17^ Usefulness of HADS is reflected by availability of more than 2834 HADS citations in the literature^9^ justifying its use in various settings though less commonly in A & E especially in the Arabian countries.

The present study evaluating depression and anxiety in hospital population using Arabic translated HADS is probably the first such endeavour in A & E setting in the Arab world. A few studies have been conducted in A & E setting in other parts of the world but on carefully selected pool of patients with lack of a study focusing general pool of A & E patients.^9^ In our study, acceptability of HADS questionnaire was quite good and only 12 subjects among 297 refused to fill. It is compatible to worldwide experience where acceptability was found to be 95% or more.^11^

In the present study, the observed occurrence of depression and anxiety was 23% and 27.2%respectively. In comparing with other similar studies, we have to appraise that the present study included all patients attending A & E as per an agreed inclusion criteria in general and did not focus on specific subsets of clinical population unlike other studies which did focus on specific pool of patients either related to their primary or main clinical complain. It did result in them reporting quite variable prevalence rates. Another arguable reason for broad variability between other studies might be due use of different cut off values for case-ness. For instance, in a Turkish study combined prevalence for both depression and anxiety was 30-50% among patient attending A & E with nonspecific chest pain (NSCP).^21^ In another but similar focused group study, the prevalence was observed as high as 73.3% among patients with NSCP using low cut off value of 8.^22^ Another study pointed out that 57% of patients without organic etiologies for their chest pain or palpitation had high HADS scores for underlying depression and anxiety.^23^ In an Australian study where the focused pool was high consumer of alcohol presented to A & E, 19.7% of the subjects were found to have depression and anxiety using high HADS cut offs.^24^

Other hospital clinic settings having stressful environment comparable to A & E like critical cardiac/coronary care (CCU), prevalence of anxiety was observed up to 24% using HADS cut off score of 10, which is very comparable to findings of the present study.^25^ In another study that did focus on general complaints though with comparative analysis of British and Australian A & E visitors, anxiety was 20.4% in British and 20% in Australian patients whereas, depression was 20% and 11% in British and Australian patients respectively.^26^ The prevalence is again comparable to the present study probably due to generalized A & E population inclusion instead of a clinical sub-group. In another comparative study in UK although focused A & E patient with serious facial trauma, 20% of the group achieved scores suggestive of depression and anxiety, both. Whereas, in a comparable sample group in Australia, 15% had case-ness for anxiety and 11.5% for depression.^27^,^28^

Its worthy to note that individual demographic variables have appeared influential in several comparable studies for instance, women found more susceptible to depression and somatization than men^29^ similar observations were observed in the present study. Furthermore, in the present study married and divorced/widowed status had high association with depression and anxiety as compared to unmarried subjects as well as low illiteracy and low socio economic status, comparably similar to other studies.^20^

### Limitations

Despite statistically significant results there are limitations in terms of over generalization as the present study included those patients who were clinically stable, thus, making the participating sample still fall short of true representation of the whole A & E population. The second limitation of the study is that the suspected cases were not provided structured psychiatric review which would have given sensitivity and specificity in terms of authentic diagnosis of depression and anxiety. However, HADS has been used and recommended without psychiatric evaluation for screening successfully.^17^

## CONCLUSION

The prevalence of depression and anxiety among patients attending A & E in a hospital setting is common and, therefore, it is appropriate to routinely use translated HADS as a screening tool for these patients. It can be a measure evolved in practice in liaison with local psychiatry services to attain not only quality but holistic patient care.
